# Comparison of Cortical Bone Fracture Patterns Under Compression Loading Using Finite Element–Discrete Element Numerical Modeling Approach and Destructive Testing

**DOI:** 10.7759/cureus.29596

**Published:** 2022-09-26

**Authors:** Nick Hudyma, Andrea Lisjak, Bryan S Tatone, Hillary W Garner, Jeffrey Wight, Akhil S Mandavalli, Ifeloluwa A Olutola, George G. A Pujalte

**Affiliations:** 1 Civil Engineering, Boise State University, Boise, USA; 2 Rock Mechanics, Geomechanica Incorporated, Toronto, CAN; 3 Civil Engineering - Rock Mechanics, Geomechanica Incorporated, Toronto, CAN; 4 Radiology, Mayo Clinic, Jacksonville, USA; 5 Kinesiology, Jacksonville University, Jacksonville, USA; 6 Family Medicine, and Orthopedics and Sports Medicine, Mayo Clinic, Jacksonville, USA; 7 Family Medicine, Mayo Clinic, Jacksonville, USA

**Keywords:** modeling, fdem, compression, fracture, bone

## Abstract

Finite element analysis may not be the only method by which bone fracture initiation and propagation may be analyzed. This study compares fracture patterns generated from compression testing of bone to fracture patterns generated using a combination of both the finite element method (FEM) and discrete element method (DEM) as defined by the finite discrete element method (FDEM). Before testing, a three-dimensional bone model was developed using CT. Force and displacement data were collected during testing. The tested specimen was reimaged using CT. The solid model was discretized and material properties adjusted such that finite element-discrete element macro behavior matched the force-displacement data. A qualitative comparison of the fracture patterns demonstrates that FDEM can successfully be used to simulate and predict fracturing in bone, with this study representing the first time this has been done and reported.

## Introduction

Stress fractures are particularly common and problematic in athletes and military personnel [1]. The incidence of stress fractures in the general population is less than 4% but can be as high as 64% in the military [2-4]. Among athletes, distance runners and track and field athletes are the most susceptible to stress fractures [5]. Around 20% of distance runners are said to have had bony stress injuries [6-7]. In track and field athletes, the incidence has been reported to be 3%-25%, with female athletes being more affected [8-11]. The tibia is the most common location for stress fractures in both athletes [12-13], and military personnel [4, 14]. As such, there is a great need to understand the pathophysiology of stress fractures, since the frequencies remain high, and the injuries are debilitating. Additionally, knowledge and understanding of tibial stress mechanics and how they propagate are important. 

Computer modeling is commonly used to study how bone deforms and fractures under applied forces. The scientific community has embraced the continuum-based finite element method (FEM) as a way of analyzing bone deformities and fractures. This method has been shown to be excellent at determining bone strength [15] and identifying highly stressed locations in the bone where fracturing may occur during loading [16]. FEM has also been used to study the simulation of fracture initiation [17-18]. However, it is possible that FEM can be improved on by incorporating the discrete element method (DEM). Rather than having a discretized continuum to stimulate stress and deformation in materials as designated by FEM, DEM represents the object under investigation using particles, unbonded or bonded, whose changing behaviors and contact points are accounted for within the model’s calculations [19]. Using the combination of FEM and DEM in the form of a finite discrete element method (FDEM) can integrate new information; FDEM incorporates local interaction and motion between the discretized particles of certain materials as determined by DEM (such as bone) to global properties of those materials as found by FEM (such as deformability of the bone) [20]. The use of both FEM and DEM in combination allows the model to accurately simulate fracture initiation and propagation of materials placed under stress, even when a strength threshold is surpassed and failure occurs [20]. 

To our knowledge, FDEM has yet to be used to investigate bone fractures. However, this technique has been used extensively in rock mechanics to investigate fracture initiation and propagation within brittle materials [21] and to assess the re-initiation of pre-existing fractures within a fracture network in a rock mass [22]. Given the success of the technique in rock mechanics, a study investigating its use for the deformation and fracture of bones is warranted. In a biomedical context, the pristine (undamaged) condition would represent a healthy bone. A bone with pre-existing (healed) stress fractures could be represented by a fracture network. 

The purpose of this study was a proof of concept to qualitatively assess how well FDEM simulation could predict laboratory bone fracture patterns generated during quasi-static compression testing of a bovine tibia. The secondary purpose was to determine whether FDEM can replicate the location of fracture when the bones were subjected to quasi-static axial force to cause failure.

## Materials and methods

This study was deemed exempt by the Mayo Clinic Institutional Review Board. The study used three teams for characterizing and comparing experimentally and numerically derived fractures. Although the teams worked together to develop and perform the study, there were portions of the study where only limited information was exchanged between teams to eliminate biases. The ASTM International C39/C39M-18 protocol was used in accordance with a standardized test method for the sample, and the ASTM International D4543-19 protocol was followed in accordance with the preparation of the test sample [23-24].

Bone sample source and preparation

A fresh bovine tibia was purchased from a local butcher. The bone specimen sample was then cut from the midshaft of the tibia, in the research laboratory, using a bandsaw. The length-to-diameter ratio of the tibia specimen was approximately 2 to 1, which is the typical dimension for testing concrete and rock under compressive stress conditions [23-24]. Similar dimensions have also been used for compression testing of bone [25]. The cut specimen was then boiled and soaked in bleach to remove all non-osseous tissue (ligament, marrow, and muscle tissue). 

Bone specimen grinding was then performed to make the specimen’s ends flat and parallel. This was done by mounting the specimen in a V block, and the ends were ground flat and parallel using a surface grinder. If the specimen’s ends were not prepared properly, premature shear failure would occur at the loading interfaces, and failure patterns would reflect shear failure rather than failure from compression loading [23]. Last, heat shrink plastic was applied to the specimen to prevent damage to test instrumentation and keep the pieces of the failed specimen together for post-test analyses. 

Pretest scanning

After preparation, the specimen was imaged in a SOMATOM Definition Flash (Siemens AG, Munich, Germany) CT scanner using the bone high-dose setting. DICOM (Digital Imaging and Communications in Medicine) image resolution was 600 × 600 ppi, and slice thickness was 2 mm. The two-dimensional DICOM images were imported into Simpleware (Synopsys, Inc., Mountainview, CA) to produce solid three-dimensional models of the cortical bone. Using the same software, the three-dimensional models were discretized and meshed using tetrahedral elements. Mesh refinement was conducted in Trelis 17.0 (Csimsoft, American Fork, UT).

Compression testing

The specimen was tested under strain-controlled compression conditions at a strain rate of 1 x 10^-5(mm/mm per s). The specimen was placed between platens for loading; the lower platen was fixed, and the upper platen had a spherical seat. The spherical seat helped compensate for malalignment within the testing frame and irregularities in the surface being loaded. Axial deformations were measured using two linear variable differential transformers supported by rings mounted to the specimens. Specimens were tested for failure.

Post-test scanning

Post-test scanning was conducted using an inspeXio SMX-225CT FPD Plus (Shimadzu Corporation, Kyoto, Japan) industrial CT scanner. Image resolution was 1200 × 1200 ppi, and slice thickness was 0.1 mm. The increased resolution was needed for accurate qualitative descriptions of the fracture patterns in the tested bone specimens.

FDEM simulation

**Figure 1:**
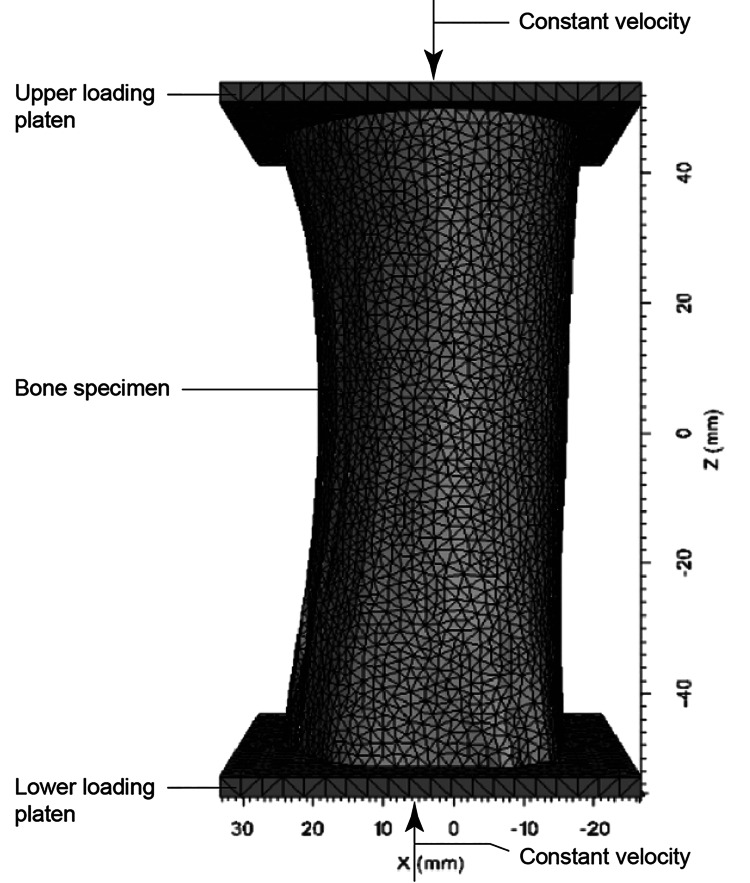
Geometry and boundary conditions of compression test simulation on bone specimen. The z-axis and x-axis are denoted through Z (mm) and X (mm) respectively. mm denotes the length of the dimensions of the bone and platens in millimeters.

The modeling team received the following data: a three-dimensional discretized and meshed model of the bone specimen, as described above, and load-deformation data from the compression testing. The 100-mm-long specimen was discretized with a tetrahedral finite element mesh having a nominal edge length of 2 mm for a total of approximately 58,000 finite elements (Figure 1). The specimen was loaded by two rigid platens moving at a constant speed in opposite directions, and platen reactionary forces were used to compute the load applied to the specimen. The modeling team did not receive any descriptions or images of the fractures generated from compression testing to avoid bias in the modeling efforts. 

In this study, Irazu 4.0 (Geomechanica Inc., Toronto, CA), a graphics processing unit-accelerated, three-dimensional, FDEM-based computed code, was used [26-27].

Calibration of numerical model

**Figure 2:**
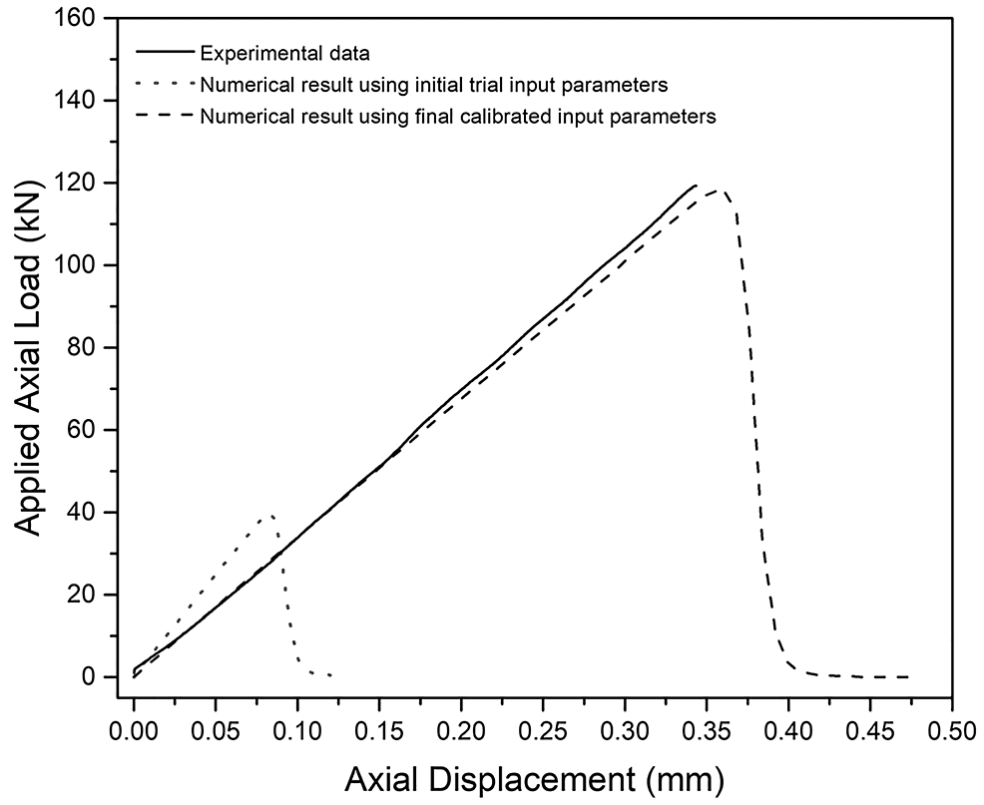
Comparison of numerical and experimental load displacement curves. mm denotes the length in millimeters and kN denotes the force of the axial load in kilonewtons.

Calibration of the numerical model for the single specimen followed a trial-and-error approach that is commonly used in discontinuum-based numerical simulations [28]. In this scenario, the parameters of the model are compared and then adjusted in response to relevant changes in the physical specimen. In this study, the calibration consisted of adjustments to micromechanical input parameters to provide the best match of a selected experimental curve of axial load vs axial displacement (Figure 2). The initial load-displacement curve (Table 1) underestimated peak load and overestimated axial stiffness of the bone specimen (Figure 2). Therefore, adjustments were made in subsequent modeling iterations. Specifically, Young’s modulus of the bone material was decreased, while tensile and shear strength parameters were increased, until a satisfactory match of both emergent axial stiffness and strength was obtained (Figure 2). Poisson’s ratio and internal friction angle values were assumed equal to 0.25 and 0.30, respectively. Shahar et al. [29], reported Poisson’s ratio values of bone from previous studies ranged between 0.12 and 0.63, and finite element analyses often use Poisson’s ratio values between 0.28 and 0.33 [30]. The assumed friction angle is between two extremes of values determined from previous studies. At the high end, friction angles of 45° were determined through finite element simulation of indentation tests using the Drucker-Prager constitutive model [31-32]. At the low end, friction angles of 15° were determined by Tai et al. and Wang et al. [33-34]. Poisson’s ratio and internal friction angle values were not subjected to numeric calibration as experimental measurements of radial displacement and the effect of confining pressure on bone strength were not available.

**Table 1:** Calibration of input parameters of the numerical model: initial and final set of values.

Parameter	Initial trial input parameters	Final calibrated value
Young’s modulus (GPa)	50	34
Poisson’s ratio	0.25	0.25
Tensile strength (MPa)	1.5	5.5
Cohesion (MPa)	10	35
Mode I fracture energy (J/m^2^)	1	11
Mode II fracture energy (J/m^2^)	10	110
Internal friction angle	30°	30°

## Results

**Figure 3:**
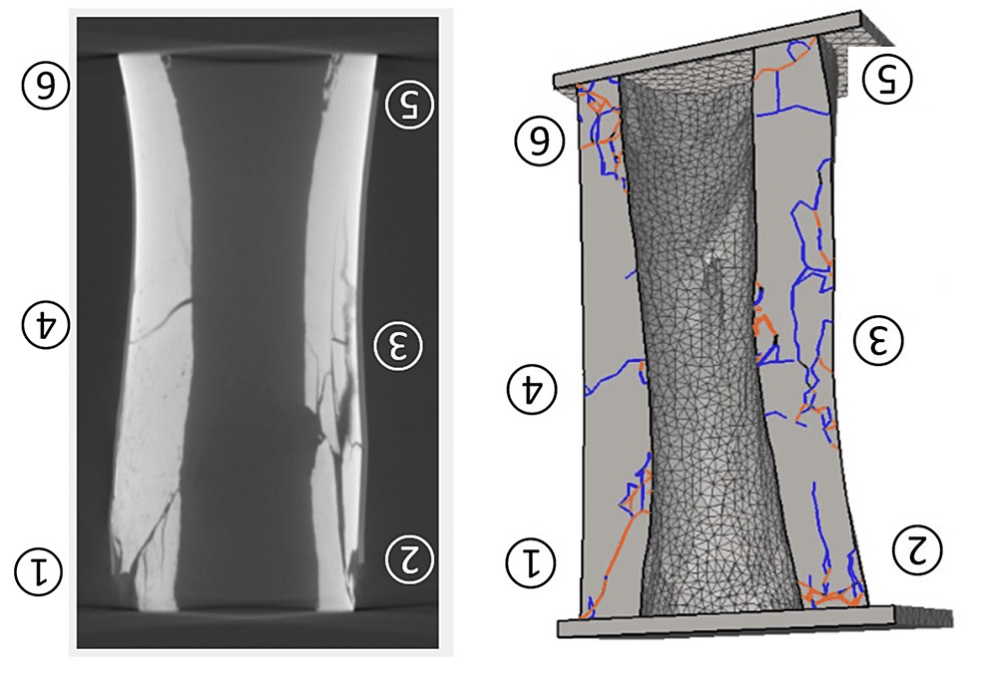
Identified regions of fractures from FDEM simulation and test specimen. Regions of interest are numbers 1-6 and are described in the text. FDEM, finite discrete element method

Figure 3 presents fracture patterns generated from the FDEM simulation vs the fracture patterns as generated from the compression loading of a single specimen (as seen by CT). For the fractured specimen and simulation results in Figure 3, six regions of interest were identified. Discussions focusing on each of the regions are presented below.

Region 1

Region 1 contained an inclined shear fracture. In the simulation, the external outside end of the shear fracture was at the interface of the bone and the loading platen. The internal inside end of the shear fracture terminated in a highly fractured zone consisting of both shear and tensile fractures. In the test specimen, the external end of the shear fracture could not be located because a piece of the specimen became detached. The internal end of the shear fracture did not appear to terminate in a highly fractured zone as in the simulation. In the test specimen, vertical tension fractures parallel to the loading direction terminated on the shear fracture, indicating the tension fractures formed before the shear fracture.

Region 2

Region 2 contained vertical tension fractures approximately parallel to the loading direction. The simulation showed tensile fracturing with minor shear fractures near the top of the bone. There were a number of small fractures in the test specimen near the proximal external portion of the bone. Additionally, a piece of bone detached from the specimen. 

Region 3

Region 3 was a highly fractured zone in both the FDEM simulation and test specimen. The simulation showed tensile fracturing with minor shear fractures on the inside and outside of the bone. The intersecting fractures shown in the simulation indicated the bone broke into smaller pieces. The test specimen clearly showed the intersecting fractures and the resulting damage to the bone, which broke into smaller pieces. In the specimen, some of the pieces rotated, indicating movement along the fractures. Both the simulation and the specimen showed vertical, sub-vertical, and horizontal fractures. The transition between Region 2 and Region 3 was different between the simulation and specimen. In the simulation, the main vertical fracture in Region 2 terminated before it reached the highly fractured zone of Region 3. In the specimen, the main vertical fracture terminated in the highly fractured zone.

Region 4

The highly fractured zone of Region 3 had a direct correlation with the subhorizontal fracture in Region 4, which resulted in buckling within Region 3. The rotation and dislocation of bone pieces in an outward direction created tensile forces in Region 4, leading to the subhorizontal fracture. 

Region 5

Regions 5 and 6 were different in the simulation and specimen. The specimen showed no discernible fractures in these regions. There should be fracturing because the specimen was placed on a rigid base during testing. The lack of visible fractures may suggest hairline fractures that could not be easily detected in the CT image. 

In Region 5, the simulation showed a horizontal tensile fracture above a subhorizontal shear fracture. The horizontal fracture formed from the tensile forces generated by the outward buckling in Region 3. The subhorizontal shear fracture formed in conjunction with the shear fracture in Region 1. 

Region 6

The fracture patterns in Region 6 were also similar to those in Region 2. As the shear fractures were forming in Regions 1 and 5, the same crushing at the proximal and distal ends of the specimens (indicated by the shear fractures) and formation of tensile fractures occurred in Regions 2 and 6.

## Discussion

For this study, a direct comparison of fracture patterns of stress fractures generated by FDEM simulation to those generated by compression loading of specimens was completed. To address the need for further research on stress fracture injuries in the US military, Jones et al. recommended a systematic, data-driven, evidence-based approach to explicitly investigate the geometry and properties of the cortical bone of the tibia [35]. The research directives included: 1) a comprehensive literature review to develop a database of bone properties; 2) a review of radiographic and CT imaging of tibiae affected by stress fractures to determine fracture characteristics; 3) laboratory testing of bone with pre- and post-CT imaging; and 4) the use of computer simulation tools specifically designed to replicate the fracture process in brittle materials [35]. 

This study followed these systematic, data-driven, evidence-based directives as outlined by Jones et al. [35]. As demonstrated in Figure 3, a qualitative comparison revealed that the fracture patterns as captured by the CT imaging showed strong similarities to the material model used for FDEM, with the most striking similarities occurring at the top and midpoint of the bone specimen. This clear qualitative association between the quasi-static compression laboratory testing of bovine tibiae and FDEM simulations of the same bone geometry indicated that FDEM can simulate fracture formation and can successfully replicate fracture locations and patterns within the tested specimen. To the study team’s knowledge, this study is the first to use FDEM to simulate both deformation and fracturing processes in bone. 

The success of such modeling suggests promise for the development of a personalized, preventative, risk-based approach to the prediction of tibial stress fractures based on FDEM as an alternative to finite element analysis (FEA). Indeed, the capacity of the FDEM model used to predict discrete fracture location and propagation shows key benefits over the use of FEA in similar studies. Ota et al. (1999) used FEM and physical experiments to investigate fracturing in femurs. The predicted fracture zones were located medial to the experimental fracture [18]. Similarly, finite element analyses predicting fractures in human femora effectively determined particular zones where applied stresses were greater than bone strength, modeling the onset of fractures but not fracture propagation [36]. Wong et al. (2010) used FEM to investigate fracture locations and patterns in a human tibia [37]. This study evaluated the maximal nodal Von Mises stresses in the cortical bone under various loading cases, including axial compression, using FEM analyses. The FEM analyses were only able to show a zone of potential fractures without particular designation of individual fractures [37]. Hambli et al. (2012) used a finite element model based on continuum damage mechanics to predict fracture patterns in femurs [17]. Their model was calibrated using data from a previous study and their predicted fracture patterns were also compared to fracture patterns seen in patient radiographs from a previous study. They were able to successfully simulate the fractured area, but not discrete fractures, of the femur [17]. These studies clearly show that while finite element analyses are able to identify general zones of probable fracturing, they are largely unable to capture individual fracture initiation and propagation within the bone as seen with the use of an FDEM model. The use of FDEM within this study also expands upon the current use of the DEM for investigating bone properties and characteristics. The DEM analysis has been shown to successfully simulate the constituent parts of a lumbar spine and their elastic response, but not fracture propagation or initiation [38]. 

Although this study was completed using a bovine tibia, animal studies are valuable in that they facilitate the design of human studies, allow for adjustments based on lessons learned, and help determine the feasibility of methodologies before humans are involved. Previous studies investigating tibial fracturing propagation in rabbit tibia have demonstrated that the quasi-static compression test utilized within this study could potentially be responsible for tibial stress fractures as physical exertions such as running can be modeled by both static and quasi-static loads [39]. Additionally, bovine tibial bone has been studied before to determine bone characteristics that may be useful in human studies [40-42]. Therefore, further investigations of bovine tibial bone geometry and stress fracture characteristics may be helpful in predicting how human tibia geometry will respond to stresses and proceed into stress fracture states when subjected to repetitive forces.

This proof-of-concept study was limited to the testing of one specimen but demonstrated successful use of the finite discrete element method (FDEM) to simulate fracture initiation and propagation in bone. Limitations also involve the preparation of the bovine tibia used, as it was boiled and soaked in bleach to remove all non-osseous material. Doing so may have altered the properties of the bone such that levels of mineralization and hardness were reduced, affecting calculated values of axial stiffness and strength [43-44]. Such physiological changes might have been contained in the underestimated peak load and overestimated axial stiffness of the bone specimen as indicated by the initial trial parameters of the calibration of the numerical model, but further analyses must be sought out. Further investigations may also adopt mineral/density testing of bone prior to procedures in consideration of this issue, and to establish a baseline of physiological parameters more effectively. Additionally, the use of heat shrink-wrap may have also changed the boundary conditions and properties of the bone such that the natural pattern of shattering was affected. The results presented within do not reflect fractures and fracture patterns that may be generated in fresh bone with non-osseous materials intact, as the use of heat-shrink wrap and boiling was necessary to load and prepare the sample for testing. 

## Conclusions

In this proof-of-concept case study, FDEM simulation was shown to effectively simulate fracture generation on a bovine tibia. The fractures and fracture patterns produced by the FDEM simulation were very similar to the fractures and fracture patterns produced through compression testing. These preliminary results suggest that the FDEM approach may enhance current simulation models.
